# Improving stand-to-sit maneuver for individuals with spinal cord injury

**DOI:** 10.1186/s12984-016-0137-6

**Published:** 2016-03-15

**Authors:** Sarah R. Chang, Mark J. Nandor, Rudi Kobetic, Kevin M. Foglyano, Roger D. Quinn, Ronald J. Triolo

**Affiliations:** Department of Veterans Affairs, Advanced Platform Technology Center, Louis Stokes Cleveland VA Medical Center, 10701 East Blvd, 151AW/APT, Cleveland, OH 44106 USA; Department of Biomedical Engineering, Case Western Reserve University, 10900 Euclid Avenue, Cleveland, OH 44106 USA; Department of Mechanical Engineering and Aerospace Engineering, Case Western Reserve University, 10900 Euclid Avenue, Cleveland, OH 44106 USA; Department of Orthopaedics, Case Western Reserve University, 10900 Euclid Avenue, Cleveland, OH 44106 USA

**Keywords:** Stand-to-sit, Knee damping, Hip-knee coupling, Electrical stimulation, Biomechanics, Sitting impact force, Hybrid neuroprosthesis, Spinal cord injury, Orthotic knee mechanisms

## Abstract

**Background:**

Users of neuroprostheses employing electrical stimulation (ES) generally complete the stand-to-sit (STS) maneuver with high knee angular velocities, increased upper limb support forces, and high peak impact forces at initial contact with the chair. Controlling the knee during STS descent is challenging in individuals with spinal cord injury (SCI) due to the decreasing joint moment available with increased knee angle in response to ES.

**Methods:**

The goal of this study was to investigate the effects of incorporating either (1) a coupling mechanism that coordinates hip and knee flexion or (2) a mechanism that damps knee motion to keep the knee angular velocity constant during the STS transition. The coupling and damping were achieved by hydraulic orthotic mechanisms. Two subjects with SCI were enrolled and each served as their own controls when characterizing the performance of each mechanism during STS as compared to stimulation alone. Outcome measures such as hip-knee angle, knee angular velocity, upper limb support force, and impact force were analyzed to determine the effectiveness of the two mechanisms in providing controlled STS.

**Results:**

The coordination between the hip and knee joints improved with each orthotic mechanism. The damping and hip-knee coupling mechanisms caused the hip and knee joint ratios of 1:1.1 and 1:0.99, respectively, which approached the 1:1 coordination ratio observed in nondisabled individuals during STS maneuver. The knee damping mechanism provided lower (*p* < 0.001) and a more constant knee angular velocity than the hip-knee coupling mechanism over the knee range of motion. Both the coupling and damping mechanisms were similarly effective at reducing upper limb support forces by 70 % (*p* < 0.001) and impact force by half (*p* ≤ 0.001) as compared to sitting down with stimulation alone.

**Conclusions:**

Orthoses imposing simple kinematic constraints, such as 1:1 hip-knee coupling or knee damping, can normalize upper limb support forces, peak knee angular velocity, and peak impact force during the STS maneuvers.

## Background

Individuals paralyzed by spinal cord injury (SCI) can achieve functional sit-to-stand, standing, stepping, and stand-to-sit (STS) maneuvers by employing different technologies such as electrical stimulation (ES), passive lower extremity orthoses, powered lower extremity orthoses commonly known as exoskeletons, or hybrid neuroprostheses. ES delivered to the peripheral motor nerves can cause the associated muscles to contract, and in so doing restore various lower extremity functions by coordinating limb movements. However, ES can rapidly fatigue deconditioned muscles, compromising the ability to complete repetitive movements or maintain the joints in a stable posture for long periods [1, 2]. Passive lower extremity orthoses can support an individual in standing but do not necessarily provide power for coordinating limb movements to walk [3, 4]. The motors used in robotic exoskeletons are responsive and can generate consistent joint trajectories. The disadvantage of the powered exoskeletons is that they do not provide the benefits of actively contracting the user’s muscles, while advantages are that they support an individual in standing and coordinate limb movements for walking [5–10]. The hybrid approach combines the health benefits of contracting the large lower extremity muscles via ES with the support and stability of lower extremity braces to facilitate standing or walking [11–14].

The stand-to-sit transition requires eccentric contractions of the lower limb extensor muscles, such as the quadriceps. While eccentric contractions can be generated with stimulation alone, controlling the knee during the STS descent is challenging in SCI due to diminished joint moment with increased knee angle in response to stimulation [15].

Various methods of sensor-based closed-loop control of ES have been developed and resulted in significant improvements to the STS maneuver. A switching curve controller for ES-assisted standing up and sitting down was designed to turn stimulation on or off depending on the knee joint angle and angular velocity. If the knee angular velocity was above the curve of nondisabled angular velocities at low or high knee angles during sitting down, stimulation would be turned on to slow the maneuver. Use of ES and the switching curve controller was shown to greatly reduce the hand-support forces but needed improvements in controlling the ending knee angular velocities [16]. In addition, a “patient-driven motion reinforcement” strategy where movement was initiated by the voluntary upper body forces and stimulation determined from an inverse dynamics model maintained movement and minimized upper body effort compared to trials without stimulation [17]. Although the controller was implemented, the subject was partially supported by a seesaw with a counterweight. In another example, a simulation study investigated a nested control strategy to support standing up and sitting down with quadriceps muscle stimulation, where the inner loop consisted of a proportional integral derivative controller and the outer loop used virtual reference feedback tuning, a feedback controller that does not require a model of the system for controller tuning. The simulation results suggested that the virtual reference feedback tuning strategy would be effective in controlling ES-based standing up and sitting down for individuals with paraplegia but lacks actual implementation in individuals with SCI [18]. Kumar et al. discussed the use of knee angle feedback with a four-channel stimulator to assist an individual with complete paraplegia in performing sitting and standing function using quadriceps and glutei muscles. The subject was able to successfully stand up, stand, and sit down with the feedback stimulation system [19]. Another technique for controlling the STS transition included a closed-loop controller using ES and joint kinematics by bilaterally instrumenting the knee joint with electrogoniometers or gyroscopes [20]. For example, the controller would turn on, turn off, or modulate the stimulation pulse width depending on the knee angle and angular velocity while the individual performed the STS transition. Using the closed-loop controller to complete the STS maneuver reduced the end velocity of the knee from 106.9 °/s to 67.6 °/s and reduced upper limb support forces to less than 50 % of the body weight. Many of the controllers described above currently provide options for controlling the STS transition with ES alone. Sliding mode and other nonlinear feedback controllers have also been explored to modulate the concentric and eccentric contractions of the quadriceps muscle and could potentially be used to control the STS transition with ES only or in combination with orthotic mechanisms [21–25]. The orthotic mechanisms evaluated in this study aim to complement these ES controllers, or offer alternative mechanical fail-safe systems if ES is unavailable or the user’s muscles have fatigued significantly to compromise controller performance.

A knee-extension assist was designed to provide a knee extension moment for a knee-ankle-foot orthosis to assist users with sit-to-stand and stand-to-sit transitions [26]. The device had three parallel compression springs opposed to a larger single spring combined with a cable system that would wrap around a knee disk via pneumatic actuator to generate tension. Two nondisabled individuals successfully evaluated the device for its ability to assist during stand-to-sit and sit-to-stand maneuvers. The knee angular velocities using the knee extension assist were not significantly lower than having no assistance for the nondisabled subjects. These initial assessments of the effectiveness of the device during the STS maneuver with nondisabled volunteers were not encouraging, and its performance in individuals with weak or paralyzed knee extensors has yet to be reported.

Moreover, none of these studies reported the impact force when the participants initially made contact with the seating surface. In individuals with SCI who lack normal sensation, repeated high impact forces, as can occur during STS [27] should be avoided to minimize the potential for injury. Deep muscular hemorrhaging can take place when impact forces cause underlying vasculature damage [28]. While such complications have not been reported with ES users, the potential risk of hemorrhage, deep tissue injury, and other pressure and impact related adverse events are possible with SCI who lack normal sensation or have compromised tissue oxygenation. Depending on the magnitude and frequency of impact with the seating surface at the end of the STS, the potential still exists for the skin, deep tissue, and vasculature injury near the ischial tuberosities which would have serious consequences to overall health.

Nondisabled individuals performing the STS maneuver exhibited an approximate 1:1 hip to knee joint angle ratio and relatively constant knee angular velocity of 84.9 ± 27.0 °/s [27]. Individuals with SCI using ES required almost twice the amount of time to complete the maneuver than nondisabled controls, due to passively extended knees with stimulation ramping off, thus delaying initiation of the STS transition [27]. In addition, neuroprosthesis users with SCI had STS maneuvers characterized by poor coordination of the hip and knee joints, increased peak knee angular velocities up to twice nondisabled individuals, increased peak upper limb support forces that averaged 3.5 times those of nondisabled controls, and higher peak impact forces at initial contact with the seat that averaged 1.4 times body weight and averaged twice that of nondisabled individuals [27]. Thus, the descent to the seat in those with SCI needs to be better controlled to reduce the impact force and reduce the upper limb effort, in order to normalize the STS transition.

The main objective of this study was to design and evaluate two different orthotic mechanisms that can be used by individuals with paraplegia to control the STS transition. It was hypothesized that adding a hydraulic knee mechanism to the exoskeleton would result in a STS maneuver more closely resembling that of nondisabled controls. In this study, two hydraulic orthotic mechanisms were designed to control the stand-to-sit (STS) transition to (1) couple knee flexion with ipsilateral hip flexion in a 1:1 ratio and (2) provide damping at the knee for constant knee angular velocities. The hip and knee joint kinematics, upper limb support forces on the walker and the impact force at the initial contact with the chair were measured to evaluate the ability of the orthotic mechanisms to improve the STS maneuver for individuals with paraplegia as compared to sitting with stimulation alone.

## Methods

### Hydraulic orthotic mechanism design

#### Hip-knee coupling mechanism

**Fig. 1:**
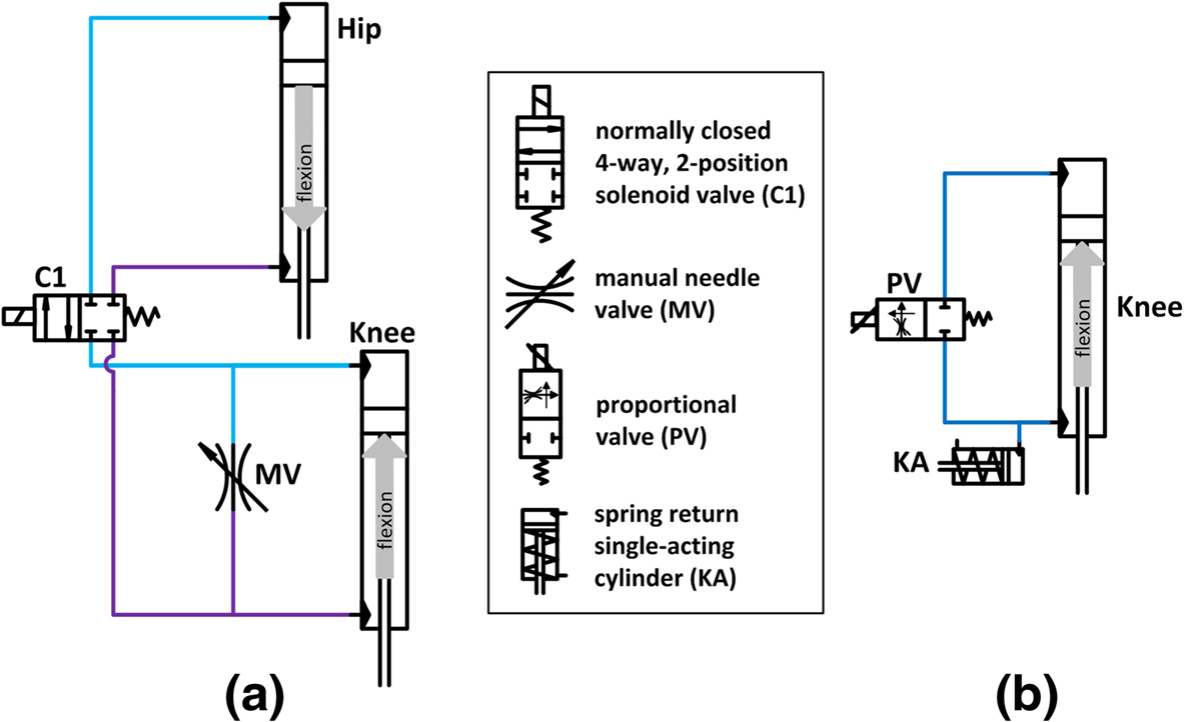
Schematics for hydraulic circuits used to create (**a**) coupling and (**b**) damping

A hip-knee coupling mechanism was designed to coordinate the ipsilateral hip and knee joints via a hydraulic circuit affixed to the exoskeleton uprights. The hydraulic circuit provided three states: locked, coupled, or uncoupled by electronically controlling the valves and directing hydraulic fluid to the appropriate locations (Fig. 1a). In this mechanism, hip flexion aided knee flexion to help initiate STS, and upper body effort tending to extend the hip slowed descent by resisting knee flexion. The hydraulic circuit was constructed from components listed in Table 1. The cylinders at the hip and knee were specified for the 1:1 ratio found in nondisabled STS maneuvers [27]. During the experimental trials, the mechanism was coupled continuously throughout the STS maneuver.

**Table 1:** Hydraulic components used in the coupling and damping mechanisms

	Hip and knee cylinders	Valves		Proportional valve	Knee accumulator
Manufacturer	Clippard Minimatic	Hydraforce	Parker Hannifin	Parker Hannifin	Clippard Minimatic
Type	double acting	4-way, 2-pos normally closed	2-way manual needle valve	2-way, normally closed	spring return single-acting
Bore	7/8″	−	–	–	3/4″
Port	1/8″ NPT	SAE 6	1/8″ NPT	SAE 4, 1/4" NPTF	1/8″ NPT
Stroke	3″	–	–	–	1″
Rod diameter	0.25″	–	–	–	0.25″
Voltage	–	12 VDC	–	12 VDC	–
Max operating pressure	2000 psi	3000 psi	6000 psi	3000 psi	250 psi
Spring force	–	–	–	–	3 lbs installed 6lbs compressed

#### Proportional valve damping mechanism

A proportional valve hydraulic circuit was designed to provide knee damping during the STS maneuver (Fig. 1b). The hydraulic circuit provided three states: locked, damped, or unlocked. The proportional valve was controlled by a pulse width modulated signal, where a duty cycle of 0 % (fully closed) locked the knee, a duty cycle of 100 % (fully opened) unlocked the knee, and varying duty cycles between 20–40 % (partially opened) damped the knee. The circuit was constructed of the components listed in Table 1. During the experimental trials, the damping mechanism was set to one constant level of damping for all subjects throughout the STS maneuver. The hip flexion was controlled by upper limbs, while the trunk was constrained by the thoracic corset.

#### Participants

Two individuals with SCI who had received implanted neuroprostheses for standing were recruited to perform the STS maneuver using the two different mechanisms. Subject A was 54 years old, weighed 68 kg and 174 cm tall. Subject B was 49 years old, with a weight of 64 kg and height of 168 cm (Table 2). All subjects signed consent forms approved by the Louis Stokes Cleveland Department of Veterans Affairs Medical Center Institutional Review Board (IRB #12055-H23) before participation in the study.

**Table 2:** Characteristics of subjects that participated in the STS experiments using different mechanisms

Subject	Sex	Age (yr)	Weight (kg)	Height (cm)	Injury Level	AIS	Time Since Injury (yr)	Time Since Implant (yr)
A	M	54	68	174	T7	A	31	29
B	F	49	64	168	T6	C	7	3

*AIS* American Spinal Injury Association Impairment Scale, *F* female, *M* male, *T* thoracic

#### Data collection

**Fig. 2:**
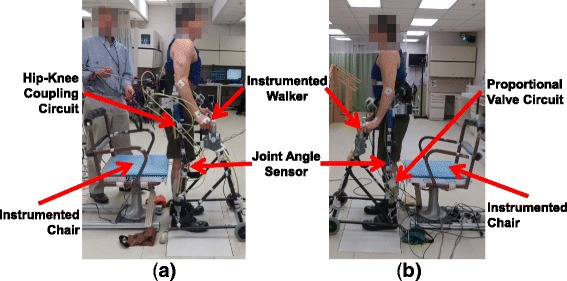
Experimental setup for an individual with SCI using the (**a**) coupling and (**b**) damping mechanism to perform the STS maneuver

Subjects with SCI performed the stand-to-sit maneuver using the two mechanisms (Fig. 2). Each hydraulic mechanism was affixed bilaterally to the uprights of the exoskeleton, with one hydraulic circuit on the right and a second of the same kind on the left upright. The brace with the coupling mechanisms weighed 16 kg and the brace with the damping mechanisms weighed 13 kg. The uprights were adjusted to fit each individual’s thigh and shank lengths, such that the anatomical hip and knee joint centers aligned with the joint centers of the brace. The thoracic corset created coupling between trunk lean and hip flexion. Subjects were able to adjust trunk lean by means of upper limbs, to affect hip flexion during the maneuver. Ankle-foot orthoses were unlocked and allowed to move freely. The subjects transferred to an instrumented chair and donned the exoskeleton. Rotary encoders (US Digital, Vancouver, WA, USA) were used to measure hip and knee joint angles. The subjects used a walker that was instrumented with load cells (AMTI, Inc., Watertown, MA, USA) at the right and left handles to measure the upper limb vertical support forces during the STS maneuver. The subjects ended the STS maneuver on a chair instrumented with a force plate (AMTI, Inc., Watertown, MA, USA) to measure the impact force. The chair was kept at the same height for both subjects. The analog data were collected at 1000 Hz.

#### Procedure

The subjects stood up by means of stimulating hip and knee extensor muscles [29] with the walker for support. The subjects initiated the STS maneuver with a finger switch which opened the hydraulic circuit valves for either coupling or damping and turned off stimulation. The STS was performed with the subjects relying on the resistance provided by the hydraulic mechanisms and their upper limb support. In the ES only condition, the stimulation of hip and knee extensor muscles ramped down. Tuning of the angular velocity with the damping mechanism was a necessary step before the subjects could successfully complete the STS maneuver without their feet slipping out from under them. The damping was empirically determined by having the subjects practice the STS transition at different damping levels. If repeated uses of a damping level resulted in the subjects’ feet slipping out from under them, that damping level was lowered. If a damping level allowed the subject to sit down slowly with feet still set on the ground, that damping level was selected for use during the experiment. The mechanism was set to the same level of damping for both subjects and kept constant throughout the entire maneuver. Similarly, the coupling mechanism was set to continuous coupling throughout the STS transition.

The participants performed four practice STS maneuvers with each mechanism before data collection took place to increase performance and reduce potential practice effects. Subjects confirmed that they were comfortable with each device and familiar with their operation, which was confirmed by observation. A 30 min rest period was provided between testing the two mechanisms to avoid potential confounding effects of testing order. Three minutes of rest in between trials minimized any potential impact of fatigue. Subjects were instructed to stand up and remain in a stable erect position, until they felt comfortable before initiating the STS. Elapsed standing times were not prescribed, but times consistently ranged from 10 to 90 s, further obviating any potential fatigue. Both subjects completed multiple trials with the coupling mechanism (Subject A: 7 trials, Subject B: 7 trials) and damping mechanism (Subject A: 5 trials, Subject B: 3 trials). The trials were not randomized, since the two different mechanisms required that the subjects doff and don the exoskeleton to switch the mechanism between conditions, which were presented in the same order to each subject.

#### Post-processing

The data were post-processed to analyze the kinematic and kinetic data. The encoder, force plate, and load cell data were filtered offline with a moving average filter (50 ms). Knee angular velocity was calculated by taking the first differential of the encoder knee angles and applying a moving average filter (50 ms). Some trials were eliminated from the analysis due to the exoskeleton hitting the chair armrests before making contact with the seating surface during the descent.

The STS maneuver started after the knee flexed by three degrees from the standing steady-state position, and ended at the impact force peak when contact was initially made with the chair [30]. The upper limb support forces and impact forces were normalized to each subject’s weight (body weight plus weight of the exoskeleton with hydraulic mechanisms).

The three conditions in the analysis were performing the STS transition using (1) ES alone, (2) the coupling mechanism, and (3) the damping mechanism. Comparisons between the different conditions were performed using Minitab 17 Statistical software (Minitab Inc; State College, PA, USA). Subjects were their own controls, such that the coupling and damping mechanism data were compared to the values measured from that same subject’s STS data with ES alone [27]. The data were analyzed using a one-way analysis of variance test (ANOVA) with Bonferroni multiple-comparison correction for a 95 % confidence interval (*p* < 0.05) to determine the statistically significant differences between the stimulation alone condition and the conditions using the mechanisms.

## Results

The mean time for Subject A to complete the STS with the coupling mechanism was 0.86 ± 0.05 s and with the damping mechanism was 1.60 ± 0.18 s (Table 3). The time for Subject A to complete the transition with the coupling mechanism was significantly less than with the damping mechanism (*p* < 0.001) or stimulation alone (*p* = 0.002), but the time was not significantly different (*p* = 0.178) when comparing the damping mechanism to stimulation alone (1.35 ± 0.31 s). The mean time for Subject B to complete the STS with the coupling mechanism was 1.58 ± 0.21 s and with the damping mechanism was 1.94 ± 0.22 s (Table 3). The time for Subject B to complete the transition with the damping mechanism (*p* = 0.004) was significantly greater than the time needed with stimulation alone (1.16 ± 0.33 s). The time to complete the maneuver with coupling (*p* = 0.05) was not significantly different when compared to stimulation alone, and the time to complete the maneuver was not significantly different (*p* = 0.2) when comparing the coupling and damping mechanisms. The mean time to complete the STS transition with the coupling and damping mechanisms were comparable to the mean time nondisabled individuals (1.51 ± 0.45 s) required for completing STS [27].

**Table 3:** Peak values for the different outcome measures recorded during the STS maneuver

	Time to Complete Maneuver [sec]		Knee Angular Velocity [deg/s]		Upper Limb Support Force [% BW]		Impact Force [% BW]	
	Subject A	Subject B	Subject A	Subject B	Subject A	Subject B	Subject A	Subject B
Stimulation Only	1.35 ± 0.31	1.16 ± 0.33	279.2 ± 26.7	154.5 ± 39.7	24.0 ± 6.2	26.8 ± 2.8	169.6 ± 27.2	164.7 ± 38.9
Coupling	0.86 ± 0.05*	1.58 ± 0.21	142.3 ± 6.3*	101.0 ± 8.2*	7.4 ± 2.8*	6.0 ± 2.2*	105.8 ± 4.2*	93.9 ± 5.2*
Damping	1.60 ± 0.18**	1.94 ± 0.22*	61.9 ± 8.6**	50.8 ± 6.0**	8.7 ± 5.4*	5.3 ± 1.9*	91.1 ± 10.7*	82.8 ± 13.1*

*Abbreviations*: *% BW* percent body weight

*p < 0.05 (mechanism condition versus stimulation only)

**p < 0.05 (coupling versus damping)

**Fig. 3:**
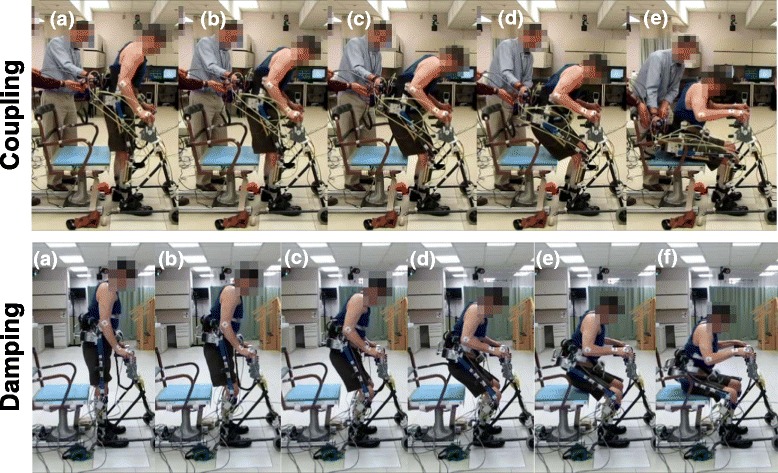
Typical progression of the stand-to-sit maneuver from quiet standing (**a**) through initiation (**b**), early and late descent (**c**-**d**) and terminal impact (**e**-**f**). Coupling (*top figure*) required controlling the hips by means of upper limbs for knee flexion which resulted in greater forward trunk movement during STS than with the hydraulic damping mechanism (*bottom figure*) where knee flexion was controlled by the mechanism alone

Both subjects began the STS with a slight forward trunk lean before the knees started to flex for both the coupling and damping conditions (Fig. 3, frame a). When using the coupling mechanism, forward trunk lean continued to increase and caused hip flexion, thereby helping break the passive knee lock that occurs at the beginning of STS (Fig. 3, coupling: frame b) [27]. As the subjects continued the maneuver with the coupling mechanism, an increase in hip flexion caused the knees to continue flexing (Fig. 3, coupling: frame c). The subjects completed the maneuver with coordinated hip and knee joints (Fig. 3, coupling: frames d-e).

When using the damping mechanism, the subjects were able to keep a more upright posture, since the hip joints were not kinematically coupled to the knees by the hydraulic circuit (Fig. 3, damping: frame b). The subjects’ upper body remained relatively erect while the knees continued to flex (Fig. 3, damping: frame c-d). The subjects showed a more upright posture with gradual hip flexion while the body was lowered by controlled knee flexion (Fig. 3, damping: frame d-f). As compared to stimulation alone [27], the STS maneuvers with the coupling and damping mechanisms had better coordinated hip and knee joints without knees locking in extension at the beginning of the maneuver and a more upright posture.

### Hip-knee angle

The knee angle at the completion of the STS transition for Subject A was 101.4° ± 0.5° and 79.2° ± 16.3° with the coupling mechanism and damping mechanism, respectively. The knee angle at the completion of the maneuver for Subject B was 89.3° ± 3.4° and 71.5° ± 6.7° with the coupling and damping mechanism, respectively. The hip angle at the completion of the STS maneuver for Subject A was 92.3° ± 6.8° and 95.0° ± 2.2° with the coupling mechanism and damping mechanism, respectively. The hip angle at the completion of the transition for Subject B was 89.6° ± 2.7° and 97.2° ± 6.3° with the coupling and damping mechanism, respectively. The final angles differed between conditions and between subjects due to variations in foot placement relative to the final sitting position and the distance between the subject and the chair before sitting down. These variables were not controlled.

**Fig. 4:**
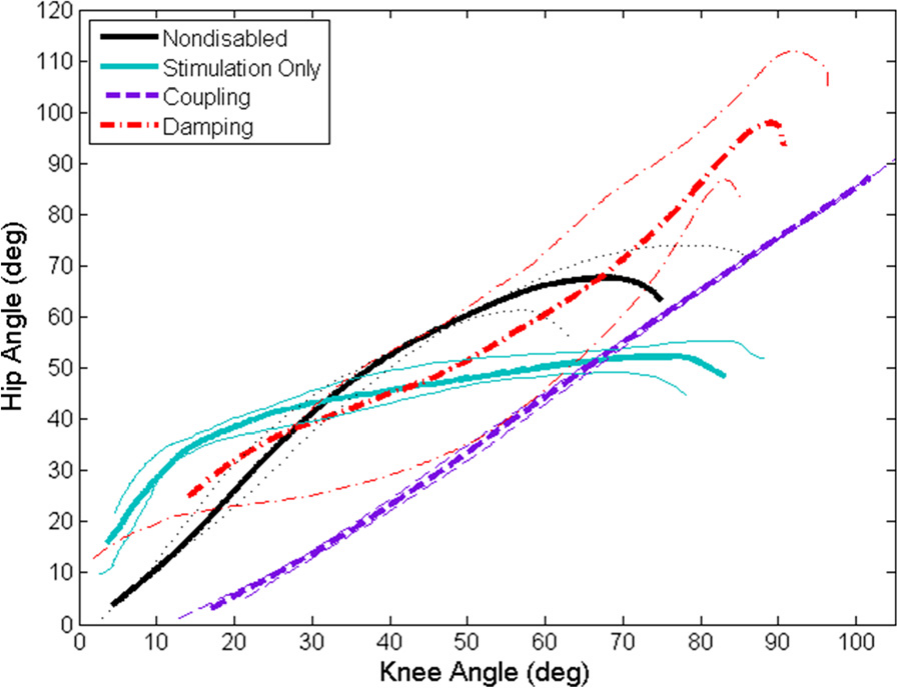
Representative average hip-knee angle and standard deviations demonstrating coordination of the hip and knee angles during the STS maneuver. Sitting with stimulation alone (*blue*) is characterized by exaggerated hip flexion at the beginning of the maneuver, followed by rapid knee flexion in the latter portion of the transition. The hip and knee angles approximate a 1:1 ratio when using the hip-knee coupling mechanism (*purple*) or the proportional valve damping mechanism (*red*) approximating that observed in nondisabled STS maneuvers (*black*) [27]

Figure 4 shows a representative hip-knee angle plot, which was fitted with a linear trendline to determine the slope of the hip-knee angle lines approximating the hip-knee angle ratio during the STS maneuver. The slope of a trendline fit to the hip-knee angle for Subject A was 0.99 ± 0.02 with *R*^2^ = 0.99 and 1.02 ± 0.28 with *R*^2^ = 0.87 for the coupling and damping mechanisms, respectively. When using stimulation alone, the slope was 0.54 ± 0.18 with *R*^2^ = 0.76 for Subject A. The slope of the trendline fitted to the hip-knee angle for Subject B was 0.98 ± 0.04 with *R*^2^ = 0.99 and 1.18 ± 0.14 with *R*^2^ = 0.95 for the coupling and damping mechanisms, respectively. When using stimulation alone, the slope was 0.58 ± 0.11 with *R*^2^ = 0.86 for Subject B. The small standard deviation of the hip-knee angle ratio suggested less variability in the coordination when using the coupling mechanism than the damping mechanism. In both subjects, there was significantly more hip flexion than knee flexion during the STS transition using ES alone. On the other hand, the hip-knee angles were more coordinated when using the orthotic mechanisms, comparable with the hip-knee angle ratios seen in nondisabled STS transitions [27].

### Peak knee angular velocity

**Fig. 5:**
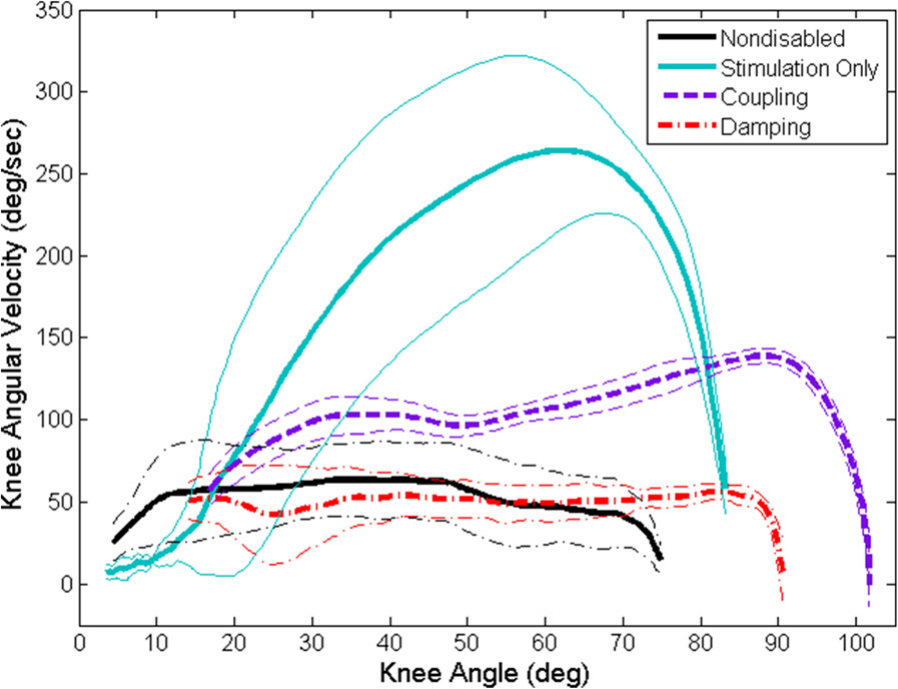
Representative average knee angular velocity and standard deviations during STS. Knee flexion angular velocity was reduced and less variable for the coupling (*purple*) and damping (*red*) mechanisms compared to stimulation alone (*blue*). Nondisabled average angular velocity indicated by black line [27]

The average peak knee angular velocity for Subject A was significantly lower (*p* < 0.001) when using the coupling (142.3 ± 6.3 °/s) and damping mechanisms (61.9 ± 8.6 °/s) as compared to the stimulation only condition of 279.2 ± 26.7 °/s (Fig. 5). Similarly, the average peak knee angular velocity for Subject B was significantly lower (*p* < 0.001) when using the coupling (101.0 ± 8.2 °/s) and damping mechanisms (50.8 ± 6.0 °/s) as compared to the stimulation only condition (154.5 ± 39.7 °/s). The peak angular velocity with the coupling mechanism was also significantly higher from that exhibited with the damping mechanism for both subjects (*p* ≤ 0.001). This difference in peak value does not necessarily mean the angular velocity over the entire range of motion of the STS maneuver for the coupling mechanism was significantly different from the damping mechanism. The knee velocities with each mechanism showed similarly shaped overall trajectories of a more constant angular velocity. However, the angular velocity with knee damping was more constant throughout the range of motion, whereas the angular velocity reached a peak near the end of the maneuver with the coupling mechanism. The coupling and damping mechanisms both showed a reduction in angular velocity and approached the angular velocities seen with nondisabled controls (84.9 ± 27.0 °/s) [27].

### Peak upper limb support force

**Fig. 6:**
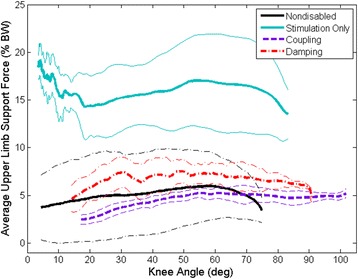
Representative average upper limb support force with standard deviation indicating a reduction in upper limb reliance when using the coupling (*purple*) and damping (*red*) mechanisms as compared to stimulation only (*blue*). Nondisabled average upper limb support force indicated by black line [27]

The average peak upper limb support force for Subject A was significantly lower (*p* < 0.001) when using either the coupling (7.4 ± 2.8 % BW) or damping mechanisms (8.7 ± 5.4 % BW) as compared to stimulation alone (24.0 ± 6.2 % BW) during STS transition (Fig. 6). Similarly, the average peak upper limb support force for Subject B was significantly lower (*p* < 0.001) when using either the coupling (6.0 ± 2.2 % BW) or damping mechanisms (5.3 ± 1.9 % BW) as compared to stimulation alone (26.8 ± 2.8 % BW). The average peak upper limb support forces were not significantly different (*p* = 1.000) for the two mechanisms. The coupling and damping mechanisms were similarly effective at reducing the average peak upper limb support force during the STS maneuver to that of nondisabled controls (7.2 ± 4.8 % BW) [27]. There does not appear to be any benefit of damping over coupling when looking at the need for upper limb support.

### Peak impact force

**Fig. 7:**
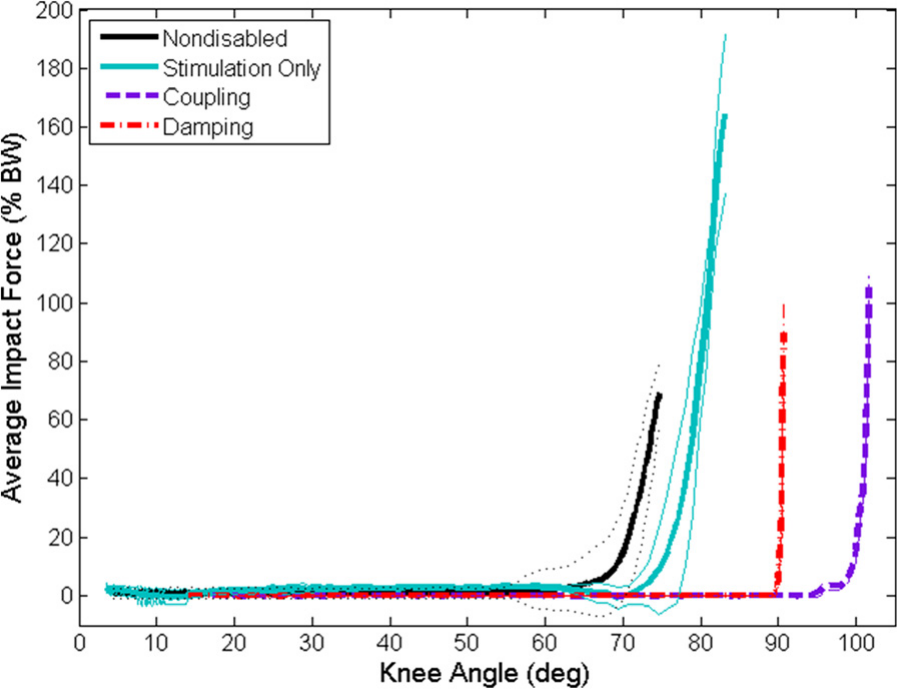
Representative average impact force and standard deviations for the different conditions indicating a reduction in the impact force when using the coupling (*purple*) or damping (*red*) mechanisms as compared to stimulation only (*blue*). Nondisabled average impact force indicated by black line [27]

Subject A had peak impact forces of 105.8 ± 4.2 % BW and 91.1 ± 10.7 % BW when using the coupling and damping mechanisms, respectively. Similarly, the peak impact forces for Subject B when using the coupling and damping mechanisms were 93.9 ± 5.2 % BW and 82.8 ± 13.1 % BW, respectively. The peak impact forces for stimulation alone were 169.6 ± 27.2 %BW and 164.7 ± 38.9 % BW for Subject A and Subject B, respectively. The peak impact force occurred when the subjects initially made contact with the instrumented chair and was on average significantly less (*p* ≤ 0.001) when using the coupling and damping mechanisms as compared to stimulation alone for both subjects (Fig. 7). The peak impact force was not significantly different for Subject A (*p* = 0.406) and Subject B (*p* = 1.000) when comparing the two different mechanisms but was approaching the lower impact forces seen with nondisabled controls (71.3 ± 9.6 % BW) [27]. Overall, there was a significant reduction in the peak impact force when using either the coupling or damping mechanism as compared to stimulation alone to complete the STS maneuvers.

## Discussion

Two subjects with paraplegia from SCI completed the STS transition using a hip-knee coupling mechanism and a knee damping mechanism. The time to complete the maneuver varied per individual. Subject A used less time to complete the maneuver using the coupling mechanism than Subject B. While not significantly different, Subject A also used less time to perform the STS with the damping mechanism. There were no explicit instructions on how fast the subjects should complete the STS, and the time to complete the maneuver using the orthotic mechanisms approached those of nondisabled controls and indicated improvement.

The coupling and damping mechanisms reduced the peak knee angular velocity to approximately 140 °/s and 60 °/s, respectively, as compared to 280 °/s when using stimulation alone for Subject A. The coupling and damping mechanisms reduced the peak knee angular velocity to approximately 100 °/s and 50 °/s, respectively, as compared to 155 °/s with stimulation alone for Subject B. The peak knee angular velocities with the coupling and damping mechanisms were also reduced when compared to the average peak value of 166.5 ± 60.3 °/s for five subjects with SCI using stimulation alone [27]. The coupling mechanism reduced the peak knee angular velocity while it still gradually increased toward the end of the maneuver. The damping mechanism helped the subjects achieve a relatively constant knee angular velocity through the maneuver which approximated that of nondisabled individuals [27].

The upper limb support forces were reduced to that of nondisabled controls (7.2 ± 4.8 % BW) and remained relatively constant, averaging approximately 6–8 %BW, throughout the STS when using the coupling and damping mechanisms (Fig. 6) [27]. The subjects were encouraged to use their arms more to control their upper body and potentially control their hips if it meant reducing the impact force at the end of the maneuver. Despite this encouragement, the upper limb support forces were significantly reduced compared to stimulation alone.

The impact force at initial contact with the chair was significantly reduced for both mechanisms as compared stimulation alone (Fig. 7). The 88–100 % BW impact forces were closer to nondisabled controls (71.3 ± 9.6 % BW), while there is still room for further reduction. Decreasing the impact force can be important for reducing the chance of injury, especially in individuals with SCI who have no sensation when sitting down on a hard surface. The damping mechanism was able to resist the impact force more than the coupling mechanism. This was expected since the damping mechanism provided a resistive torque throughout the maneuver, whereas the coupling mechanism only coordinated the hip and knee joints together but did not actively provide any damping.

In general, there was an improvement of the lower limb kinematics when the participants wore the lower extremity orthosis. For example, the subjects did not exhibit excessive forward trunk lean as was seen during STS with stimulation alone. In addition, the coordination of the hip and knee joints approached nondisabled kinematics when using the hip-knee coupling and knee damping mechanisms as compared to using stimulation alone. Use of the orthotic mechanisms did require some practice for the subjects to determine how to best complete the STS transition since the movement was different than with stimulation alone. It was challenging for the subjects to use the coupling mechanism, often because it required control of the hip joints using the walker which did not necessarily present itself in a mechanically favorable position (i.e. in front rather than behind) for helping the subjects during the maneuver.

A proper selection of damping was important to enable controlled knee flexion during the STS transition. Inhibiting the STS with a damper that is too stiff will result in a high impact force caused by the user’s feet slipping out from under them and falling backwards. It is possible to combine the damping mechanism with the coupling mechanism, however, it is important to keep the hydraulic system compact and with the least number of components as possible to minimize passive resistance. Damping has fewer components and is simpler to implement than the hydraulic circuitry for 1:1 coupling. In addition, fixed ratio coupling is likely to restrict activities other than STS, such as walking which may need modulated coupling. Knee damping mechanisms may have additional benefits during loading response to control stance phase knee flexion of gait or loading during stair descent. A hybrid neuroprosthesis designed for stepping could benefit from the coupling and damping mechanisms in this study, such as coupling hip and knee joints during early swing phase and damping during stance phase knee flexion. These mechanisms are intended to place constraints on the joints as needed while the ES provides the necessary power for standing up and initiating the step.

The differences between all average outcome measures when comparing stimulation alone to either the coupling or damping mechanism were significant for each subject and test condition. While the hip and knee joints were not well coordinated with the damping mechanism, the use of a damper at the knee during the STS appeared to be more beneficial than solely coupling the joints. However, the upper limb support forces and impact force data over the entire maneuver did not support whether the damping mechanism improved the STS better than the coupling mechanism, or vice versa. The results demonstrate that the coupling and damping mechanisms are better than stimulation only, in reducing knee angular velocity, upper limb support force, and impact force for the two participants of this study. However, generalizability of these results is limited due to small subject population. Additional experiments need to be performed and more subjects would need to be recruited to further validate and generalize the ability of the coupling and damping mechanisms to improve the control of the STS maneuver for the population of individuals with SCI. In addition, the stimulation only data were collected in a separate experimental session on a different day which places a limit on the generalizability and interpretation of the results.

The mechanisms tested in this study were all controlled with an open-loop system, where the mechanisms held their state of either coupled or damped. Future considerations of the coupling and damping mechanisms would develop a control system to close the loop. A closed-loop controller for the coupling mechanism could switch between coupled, freed or locked depending on the user’s knee angular velocity. However, this would likely compromise the smoothness of the STS transition due to energizing and deenergizing the hydraulic valves in the circuit. Closing the loop with the proportional valve damping mechanism could involve modulating the opening of the valve depending on knee angle and angular velocity during descent. Furthermore, the subjects in this study completed the STS transition with assistance from the orthotic mechanisms only. It was found that for feedforward controlled ES only STS maneuvers, subjects tended to begin descent only after stimulation had ceased [27]. Because ES only controllers have been developed and successfully implemented, the next step will be coordinating the feedback ES controllers with similar orthotic mechanisms in a future study to determine the full hybrid approach for controlling the knee during the STS transition.

## Conclusions

The results of this study demonstrated the ability to better control STS maneuvers with two different mechanisms, a hydraulic hip-knee coupling mechanism and a hydraulic damping mechanism. By incorporating a coupling or damping mechanism into a hybrid neuroprosthesis combining electrical stimulation and advanced mechanical bracing, two individuals with SCI were able to improve their STS maneuver with better coordinated joint angles, lower knee angular velocities, smaller upper limb support forces, and reduced impact forces when sitting down. By using a simple orthotic mechanism to damp or couple the joints of the lower limbs, a controlled stand-to-sit transition can be achieved in paraplegia which emulates nondisabled individuals.

## Abbreviations

% BW: percent body weight
ES: electrical stimulation
SCI: spinal cord injury
STS: stand-to-sit
